# Poor glycemic control in younger women attending Malaysian public primary care clinics: findings from adults diabetes control and management registry

**DOI:** 10.1186/1471-2296-14-188

**Published:** 2013-12-10

**Authors:** Ai Theng Cheong, Ping Yein Lee, Shariff-Ghazali Sazlina, Bujang Mohamad Adam, Boon How Chew, Ismail Mastura, Haniff Jamaiyah, Syed-Abdul-Rahman Syed Alwi, Taher Sri Wahyu, Mat-Nasir Nafiza

**Affiliations:** 1 Department of Family Medicine, Faculty of Medicine and Health Sciences, Universiti Putra Malaysia, Serdang, Selangor 43400, Malaysia; 2 Biostatistics Unit, 1st floor MMA House, Jalan Pahang, Kuala Lumpur 50586, Malaysia; 3 Seremban 2 Health Clinic, Jalan S2 A2 Seremban 2, Seremban 70300, Negeri Sembilan, Malaysia; 4 Department of Family Medicine, Faculty of Medicine and Health Sciences, Universiti Malaysia Sarawak, Lot: 77, KTLD, Jalan Tun Zaidi Adrucee, Kuching 91350, Sarawak, Malaysia; 5 Simpang Kuala Health Clinic, Kuala Kedah Road, Alor Setar, Kedah 05400, Malaysia; 6 Faculty of Medicine UiTM, Jalan Hospital Sungai Buloh, Level 7, Academic Block, Faculty of Medicine UiTM, Sg Buloh Campus, Jalan Hospital Sungai Buloh, Shah Alam, Selangor 47000, Malaysia

**Keywords:** Type 2 diabetes mellitus, Reproductive age women, Glycemic control, Registry

## Abstract

**Background:**

Women of reproductive age are a group of particular concern as diabetes may affect their pregnancy outcome as well as long-term morbidity and mortality. This study aimed to compare the clinical profiles and glycemic control of reproductive and non-reproductive age women with type 2 diabetes (T2D) in primary care settings, and to determine the associated factors of poor glycemic control in the reproductive age group women.

**Methods:**

This was a cross-sectional study using cases reported by public primary care clinics to the Adult Diabetes Control and Management registry from 1st January to 31st December 2009. All Malaysian women aged 18 years old and above and diagnosed with T2D for at least 1 year were included in the analysis. The target for glycemic control (HbA1c < 6.5%) is in accordance to the recommended national guidelines. Both univariate and multivariate approaches of logistic regression were applied to determine whether reproductive age women have an association with poor glycemic control.

**Results:**

Data from a total of 30,427 women were analyzed and 21.8% (6,622) were of reproductive age. There were 12.5% of reproductive age women and 18.0% of non-reproductive age women that achieved glycemic control. Reproductive age group women were associated with poorer glycemic control (OR = 1.5, 95% CI = 1.2-1.8). The risk factors associated with poor glycemic control in the reproductive age women were being of Malay and Indian race, longer duration of diabetes, patients on anti-diabetic agents, and those who had not achieved the target total cholesterol and triglycerides.

**Conclusion:**

Women with T2D have poor glycemic control, but being of reproductive age was associated with even poorer control. Health care providers need to pay more attention to this group of patients especially for those with risk factors. More aggressive therapeutic strategies to improve their cardiometabolic control and pregnancy outcome are warranted.

## Background

The number of people afflicted with diabetes is increasing both in developing and developed countries with higher prevalence in men than in women [1,2]. However, there are more women with diabetes than men [2]; in some countries, women are more affected, and have an increased risk of cardiovascular diseases and mortality [3-5]. Besides this, the incidence of diabetes is increasing among the younger age groups in the developing countries, reflecting the parallel increase in the prevalence of obesity and leading of a more sedentary lifestyle [2].

Malaysia is a country with multi-ethnic population. It consists of 28.3 million population with 67.4%, Bumiputera (Malays and indigenous); 24.6% Chinese; 7.3%, Indians and 0.7%, others [6]. In West Malaysia, the Malays are the predominant ethnic group which constituted 63.1% of the population. Women represent 48.4% of the population [6]. The prevalence of diabetes in Malaysia has increased from 8.3% in 1996 to 14.9% in 2006 for those aged 30 years old and above [7]. The prevalence of diabetes among women aged 18 years and above (11.3%) was comparable to men (11.9%) [7]. As younger people become affected by diabetes, more women in the reproductive age group are also becoming affected [8,9]. Type 2 diabetes (T2D) remains the most common type of diabetes in this country [10].

Women of reproductive age and with diabetes require special attention. Apart from being vulnerable to long-term diabetes complications such as nephropathy, retinopathy, and cardiovascular diseases [4,11]; they are susceptible to high-risk pregnancy, which would affect both maternal and fetal outcomes [8,9,12]. Pregnant women with diabetes have an increased risk of developing pre-eclampsia, overt nephropathy, and proliferative retinopathy [8]. In addition, there are increased risks of an operative procedure as well as post-partum hemorrhage [12].

Glycemic control is important in diabetes management. A longitudinal study has shown the benefits of intensive glycemic control in risk reduction of microvascular and macrovascular complications and these benefits were maintained for over a decade [13,14]. In addition, for women in reproductive age group, optimum glycemic control before and during pregnancy reduces the risks of fetal complications such as congenital malformations, macrosomia, perinatal mortality as well as risks of future childhood obesity, glucose intolerance, and diabetes mellitus [15-18].

Some studies have reported that younger patients with T2D were associated with poorer glycemic control [19,20]. However, we are unable to find literature on this among T2D women in the primary care setting. We would like to know the status of glycemic control in the reproductive age women compared to the non-reproductive age women. Thus, the objective of this study was to compare the clinical profiles and glycemic control of reproductive and non-reproductive age women with T2D in the primary care settings, and to determine the associated factors of poor glycemic control in the reproductive age group women. It is hoped that the results will provide preliminary evidence on the extent of the problem in these women in primary care and to better strategize health care planning for these patients.

## Methods

### Study design and population

This was a cross-sectional study of women with T2D using the data from the Adult Diabetes Control and Management (ADCM). It is a web-based diabetes registry initiated in May 2008. A total of 70,889 T2D patients aged 18 years and above from 303 public primary care clinics and hospitals in Malaysia were registered by December 2009. All the public health clinics and hospitals that managed diabetes were invited to participate in this registry. Participation in ADCM was voluntary for the patients and the health centres. The patients were informed of the on-going registry and opportunity to opt out was provided. The details of the registry have been described elsewhere [21,22].

For our study, we extracted data of 30,427 Malaysian women with T2D from 282 public primary care clinics seen between 1st January and 31st December 2009. All patients included were women diagnosed with T2D for at least 1 year and were on treatment (diet control, oral anti-diabetic agents, and/or insulin), and on follow-up. Women who were pregnant during the period of registry entry were not registered at the outpatient clinics as they were on follow-up with the antenatal clinics. The reproductive age group of women was defined as women aged 15 to 49 years [23,24]. However, the registry only recruited adult women 18 years old and above. Thus, we defined women in the reproductive age group as those 18 to 49 years old and for women in the non-reproductive age group as those 50 years old and above. This study complied with the Declaration of Helsinki and the Medical Research and also obtained approval from the Medical Research Ethics Committee, Ministry of Health, Malaysia (NMRR-08-12-1167).

### Data collection

The details of this project’s methodology and data collection have been described in previous studies [21,22]. The data extracted for analysis for this paper included demographic data (age and race [Malay, Chinese, Asian Indian, and indigenous Bumiputera]), duration of diabetes, co-morbidity, blood pressure (BP), weight, height, body mass index (BMI), waist circumference, treatment modalities, diabetes complications, and laboratory assessments [fasting blood glucose (FBG), glycosylated haemoglobin (HbA1c), serum creatinine, albuminuria, microalbuminuria, fasting plasma total cholesterol (T. Chol), low-density lipoprotein cholesterol (LDL-C), high-density lipoprotein cholesterol (HDL-C), and triglyceride (TG)]. Results of clinical examination and laboratory assessments were accepted only if they were performed within 12 months of data collection. Imputation was not performed for missing values and missing values were excluded from analysis.

Presence of co-morbidity was defined as the presence of either concomitant hypertension or dyslipidemia or both. Hypertension was diagnosed if systolic BP was 130 mmHg and above and/or diastolic BP was 80 mmHg and above, persistently or those whose current treatment comprised anti-hypertensive agents [25]. The diagnosis of dyslipidemia was based on the presence of either one or in combination with elevation of LDL-C, TG, and/or reduction of HDL-C [26].

In this study, the outcome measure was control of glycemia. All the treatment targets were defined according to the Malaysian Clinical Practice Guideline on Management of Type 2 Diabetes Mellitus [27]. Glycemic control was achieved if the HbA1c <6.5%, and BP was targeted if ≤130/80 mmHg. Body mass index (BMI) was classified into underweight (< 18.5 kg/m^2^), normal (18.5 – 22.9 kg/m^2^), pre-obese (23 – 27.4 kg/m^2^), and obese (≥ 27.5 kg/m^2^), while the waist circumference was targeted if <80 cm. The control for lipids was defined as LDL-C ≤ 2.6 mmol/L, HDL-C ≥1.1 mmol/L, and TG ≤ 1.7 mmol/L.

The diabetes complications evaluated in this study included retinopathy, nephropathy, cerebrovascular disease/stroke (CVD), ischemic heart disease, and foot problems. The diagnosis of these complications was based on symptoms, signs, laboratory and radiological evidence, as well as treatment history made by the attending doctors at the clinics.

### Statistical analysis

Categorical variables were presented as frequency (n) and percentage (%), and numerical variables were presented as mean and standard deviation (SD) because the dataset was large and normality is assumed. No imputation was performed for missing data, therefore the denominator used in the analysis varied for each variable.

To determine whether reproductive and non-reproductive age group women have an association with poor glycemic control, both univariate and multivariate approaches of logistic regression were applied. The hypothesis of this model was to determine whether women in the reproductive group have a higher level of HbA1c compared to non-reproductive group. Fifteen variables were controlled in the analysis stage because these 15 variables were suspected as confounders that could also contribute to HbA1c status. In addition, the 15 confounders were confirmed significant at univariate analysis stage. The 15 confounders were ethnicity, duration of diabetes in years, treatment of diabetes, self-monitoring of blood glucose, statuses of BMI, blood pressure, foot problem, cerebrovascular disease, nephropathy, ischemic heart disease, concomitant morbidity, LDL, HDL, total cholesterol, and total triglyceride. Multivariate logistic regression was used to confirm whether the reproductive group still has poorer control after adjusting for all the remaining potential confounders.

To examine the risk factors that could contribute to the outcome of poor control of HbA1c among women in the reproductive age group, eight out of nine significant contributing factors of poor control of HbA1c for women were selected (race, BMI, blood pressure status, duration of diabetes, management, concomitant co-morbidity, total cholesterol, triglyceride status). We excluded CVD status in the multivariate analysis as the number was too small. There were 18 patients with CVD in the reproductive group, but out of 18, eight did not report their HbA1c; two patients had good control of HbA1c and the other eight patients had poor control of HbA1c. Therefore, we dropped CVD in the multivariate analysis to avoid invalid result. All analyses were carried out using PASW 18.0 (SPSS, Chicago, IL).

## Results

There were 30,427 women who fulfilled our inclusion criteria and they were included in the analysis, with 21.8% (6,622/30,427) in the reproductive age group. The mean age for the reproductive age group women was 43.2 (SD 5.6) years (range 18–49 years) and the mean age for the non-reproductive age group women was 62.0 (SD 8.3) years (range 50–104 years). There was higher proportion of Indian women (21.2%) in the reproductive age group than in the non-reproductive age group women (17.1%). There was higher proportion of reproductive age women with duration of diabetes for less than 5 years. A smaller proportion of women of reproductive age reported co-morbidities of hypertension and dyslipidemia as well as diabetes complications. The demographic and clinical characteristics are summarized in Table 1.

**Table 1:** Demographic, complications and co-morbidity of women with T2D in the reproductive age and non-reproductive age group

	**Reproductive**^**a **^**n (%)**	**Non-reproductive**^**b **^**n (%)**	**P-value**
Age at diagnosis^c^ (n= 30427 )	38.7 (6.2)	55.5 (9.1)	<0.001
Duration of diabetes (n=30427)			<0.001
<5 years	4115 (62.1)	10325 (43.4)	
5 – 10 years	2103 (31.8)	9600 (40.3)	
>10 years	404 (6.1)	3880 (16.3)	
Race (n= 30417)			<0.001
Malay	4597 (69.4)	14893 (62.5)	
Chinese	561 (8.5)	4663 (19.6)	
Indian	1402 (21.2)	4058 (17.1)	
Others	59 (0.9)	184 (0.8)	
Complications			
Retinopathy (n= 18678)			<0.001
Yes	203 (4.8)	1387 (9.6)	
No	4006 (95.2)	13082 (90.4)	
Ischemic heart disease (n= 22004)			<0.001
Yes	66 (1.4)	878 (5.1)	
No	4766 (98.6)	16294 (94.9)	
Cerebrovascular disease (n=23661)			<0.001
Yes	18 (0.3)	213 (1.2)	
No	5239 (99.7)	18191 (98.8)	
Nephropathy (n= 22209)			<0.001
Yes	412 (8.4)	1886 (10.9)	
No	4519 (91.6)	15392 (89.1)	
Foot problem (n=23968)			<0.001
Yes	170 (3.2)	948 (5.1)	
No	5133 (96.8)	17717 (94.9)	
Co – morbidity (n=30427)			<0.001
No	1878 (28.4)	2686 (11.3)	
Hypertension only	1766 (26.6)	7322 (30.7)	
Dyslipidemia only	947 (14.3)	1919 (8.1)	
Both	2031 (30.7)	11878 (49.9)	

^*a*^*women aged 18 to 49 years,*^*b*^*women aged 50 and above,*^c^*Reported in mean (SD)*.

There was higher proportion of reproductive age women who were obese. Furthermore, significantly fewer women of reproductive age achieved the targets of HbA1c, FBG, HDL-C, and waist circumference compared to non-reproductive age women. Almost 70% to 80% of the women (reproductive and non- reproductive age) did not achieve the target for most of the clinical parameters except for TG and HDL-C (Table 2). The majority of diabetic women (both group) used oral anti-diabetic agents and only about 10% were on insulin (alone and in combination) (Table 3).

**Table 2:** Clinical parameters of women with T2D in the reproductive age and non-reproductive age group

	**Reproductive**^**a **^**n (%)**	**Non-reproductive**^**b **^**n (%)**	**P-value**
BMI category (n=28670)			<0.001
Normal	553 (8.8)	3929 (17.6)	
Underweight	58 (0.9)	409 (1.8)	
Pre-obese	2043 (32.4)	8399 (37.5)	
Obese	3648 (57.9)	9631 (43.1)	
Waist circumference^c^ (n= 14276)	91.1 (12.3)	89.7 (12.0)	<0.001
Waist circumference status(n=14276)			0.022
Achieved	475 (15.3)	1900 (17.0)	
Not achieved	2633 (84.7)	9268 (83.0)	
Blood pressure status (n= 29885)			<0.001
Achieved	1895 (29.1)	5263 (22.5)	
Not achieved	4620 (70.9)	18107 (77.5)	
HbA1c^c^ (n=20434)	8.83 (2.21)	8.29 (2.16)	<0.001
HbA1c status (n=20434)			<0.001
Achieved	550 (12.5)	2890 (18.0)	
Not achieved	3862 (87.5)	13132 (82.0)	
Fasting blood glucose^c^ (n=20188)	9.33 (3.45)	8.49 (3.39)	<0.001
Fasting blood glucose status (n= 20188)			<0.001
Achieved	719 (16.3)	3843 (24.3)	
Not achieved	3685 (83.7)	11941 (75.7)	
Total cholesterol^c^ (n=24983)	5.33 (1.17)	5.44 (1.24)	<0.001
Total cholesterol status(n= 24983)			0.006
Achieved	2896 (53.4)	10199 (52.7)	
Not achieved	2527 (46.6)	9145 (47.3)	
HDL^c^ (n=21466)	1.30 (0.51)	1.36 (0.51)	<0.001
HDL status (n= 21466)			<0.001
Achieved	3255 (69.4)	12730 (75.9)	
Not achieved	1413 (30.6)	4048 (24.1)	
LDL^c^ (n=21275)	3.21 (1.05)	3.25 (1.11)	0.017
LDL status (n=21275)			0.391
Achieved	1335 (28.7)	4882 (29.4)	
Not achieved	3314 (71.3)	11744 (70.6)	

^*a*^*women aged 18 to 49 years,*^*b*^*women aged 50 and above,*^c^*Reported in mean (SD)*.

**Table 3:** Management of diabetes for women with T2D in reproductive age and non-reproductive age group

	**Reproductive**^**a **^**n (%)**	**Non-reproductive**^**b **^**n (%)**	**P-value**
Treatment of diabetes (n= 29871)			0.107
Diet only	90 (1.4)	277 (1.2)	
Oral ADA only	5559 (85.4)	20093 (86.0)	
Insulin only	173 (2.6)	518 (2.2)	
Both oral and insulin	687 (10.6)	2474 (10.6)	
Anti-hypertensive (n= 21953)			<0.001
1	2099 (58.6)	7971 (43.4)	
2	1053 (29.4)	6516 (35.5)	
>2	431 (12.0)	3883 (21.1)	
Lipid lowering agent (n=15620)			<0.001
Statin only	2448 (89.3)	11857 (92.1)	
Fibrate only	259 (9.4)	901 (7.0)	
Others^#^	34 (1.3)	121 (0.9)	
Self monitoring blood glucose (n= 11756)			0.485
Yes	248 (9.6)	928 (10.1)	
No	2325 (90.4)	8255 (89.9)	

^*a*^*women aged 18 to 49 years,*^*b*^*women aged 50 and above,*^#^*Refer to any combination of statin, fibrate and others, ADA* = *anti-diabetic agents*.

In examining the risk factors towards the poor control of HbA1c of patients with T2D among women, the multiple logistic regression analysis with the control of other 15 confounders showed that age group was still found to be significant. The reproductive age group women was 1.5 times (OR = 1.5, 95% CI = 1.2- 1.8) more likely to be associated with poor control of HbA1c compared with the non-reproductive group.

Further analysis showed that risk factors associated with poor glycemic control in the reproductive age women were being of Malay and Indian ethnicity, longer duration of diabetes, patients on anti-diabetic agents, and those who had not achieved the target of total cholesterol and triglycerides (Table 4).

**Table 4:** Factors associated with poor glycemic control (HbA1c ≥6.5%) among reproductive age women with T2D

	**OR**	**95% CI**	**P - value**
Race			0.001
Chinese	ref		
Malay	1.600	1.152, 2.223	0.005
Indian	1.936	1.320, 2.839	0.001
Others	0.756	0.346, 1.652	0.483
Duration of DM			<0.001
<5 years	ref		
5 – 10 years	1.567	1.250, 1.965	<0.001
>10 years	4.017	1.928, 8.369	<0.001
Treatment of diabetes			<0.001
Diet only	ref		
Oral anti-diabetic agents only	8.103	3.777, 17.382	<0.001
Insulin only	18.891	5.774, 61.808	<0.001
Both oral and insulin	46.202	17.204,124.007	<0.001
Total cholesterol			
Not achieved	1.291	1.040, 1.603	0.020
Achieved	ref		
Triglyceride status			
Not achieved	1.788	1.453, 2.199	<0.001
Achieved	ref		

Control for race, BMI, blood pressure status, duration of diabetes, management, concomitant co-morbidity, total cholesterol, triglyceride status.

## Discussions

In this study, it is shown that one-fifth of women with T2D were in the reproductive age group. This would contribute to a significant health care concern. Thus, the health care system needs to strategize the healthcare planning to be able to cater to their special needs and concerns.

According to the position statement of American Diabetes Association and the European Association for the Study of Diabetes, the reproductive age group women require more stringent glycemic control in view of younger age, longer life expectancy, shorter duration of the disease, and lesser co-morbidities and complications [28]. However, this study showed that these women had poorer glycemic control despite majority of them having diabetes for less than 5 years and without complications. After controlling for all the potential confounders, being in the reproductive age group remained a significant factor for poor glycemic control.

The social expectation of women of reproductive age is to procreate and have a family, regardless of their medical condition. Therefore, measures need to be taken for reproductive age women with diabetes to achieve good glycemic control before and during pregnancy to improve the outcome of that pregnancy [29]. The modifiable risk factors associated with poor glycemic control identified in this study were patients on anti-diabetic agents and those who had not achieved the target level of total cholesterol and triglycerides. Multivariate logistic regression showed that those with existing extensive treatment with combination of insulin and oral anti-diabetic agents were found to have poorer glycemic control. This was most probably due to confounding by indication (i.e. they were on these agents due to poor control). Another reason could be late initiation of insulin at a later stage of disease when glycemic control became difficult. Thus, use of insulin is the result of poor glycemic control. Furthermore, a study has shown that there were delays in treatment intensification with oral anti-diabetic agents and insulin in people with T2D despite suboptimal glycemic control [30]. Hence, more aggressive management such as early initiation of insulin and intensified oral treatment is needed. This should occur early after the diagnosis was made as a study showed that achieving early and intensive glycemic control has the potential to preserve pancreatic beta cell function for at least 3.5 years [31].

Our results showed that a high proportion (71.6%) of women of reproductive age with diabetes had concomitant hypertension, dyslipidemia or both. They had poorer metabolic control in most of the clinical parameters such as HbA1c, FBG, HDL-C and LDL-C and waist circumference. Furthermore, those who had not achieved the target level of total cholesterol and triglycerides were at risk of poor glycemic control. Socio-cultural behavior such as dietary habits and lifestyle play an important role in the glycemic and lipid control. Some of the barriers reported in the literature for management of diabetic women during pregnancy included difficulty of maintaining a healthy diet and regular exercise, lack of social support and barriers for insulin injection and home sugar monitoring [32]. Further studies are needed to explore the barriers and difficulties faced by women of reproductive age with T2D in local settings to address their concerns.

Preconception, prepregnancy, and contraceptive care are of utmost importance for a good pregnancy outcome and improvement of women’s quality of life [33-35]. The treatment of diabetes in women, especially in those of reproductive age, requires special consideration. The choice of anti-diabetic agents’ use must consider possible outcomes on fertility, interactions with hormonal contraception, fetal health, breastfeeding, and bone health [9,36,37].

### Strength and limitations

The strength of this study is the data from the public primary care clinics, which serves the majority of patients with T2D in this country [38]. In addition, the data were extracted from the nationwide registry which was real-life practice data that reduced selection bias. Although the sample might not cover all women in Malaysia with T2D, this result could be generalized to the care of women with T2D in primary care setting in this country due to its large sample size. A previous study found that when the sample size reached at least 500, the statistics analyzed from the sample are likely the same with the parameters in that particular population [39].

Among the limitations of this study were those inherent to studies using secondary data. Missing values in certain variables might affect the true associations of the results. Also given the fact that this was a cross-sectional study, therefore, the causal-effect relationship between poor glycemic control and its predictors cannot be truly established. Other factors include compliance, usage of hormonal pills (oral contraceptive pills, hormone replacement therapy), and the competency of the primary care physician that were not captured and could have an effect on the results.

## Conclusions

Women of reproductive age with T2D were associated with poorer glycemic control compared to non-reproductive age women. The risk factors associated with poor glycemic control in the reproductive age women were being of Malay and Indian race, longer duration of diabetes, patients on anti-diabetic agents, and those who had not achieved the target total cholesterol and triglycerides.

## Competing interests

The authors declare that they have no competing interests.

## Authors’ contributions

ATC, PYL, SGS and BHC conceptualized the analysis, interpreted the data and produced the first draft of the manuscript. IM, JH, TSW, SARSA developed the study design and managed data collection. BMA performed the statistical analysis and interpreted the data. All authors have contributed to subsequent revisions and critically revised the manuscript. All authors read and approved the final manuscript.

## Pre-publication history

The pre-publication history for this paper can be accessed here:

http://www.biomedcentral.com/1471-2296/14/188/prepub
